# Very early cardiac hemodynamic changes after atrial fibrillation ablation

**DOI:** 10.1038/s41598-025-28591-5

**Published:** 2025-12-29

**Authors:** Wanyu Hu, Shiquan Chen, Hui Cheng, Yankai Mao, Bowen Zhao, Shiyan Li, Bei Wang, Hailin Tang, Chenyang Jiang

**Affiliations:** 1 Department of Diagnostic Ultrasound and Echocardiography, Sir Run Run Shaw Hospital, Zhejiang University School of Medicine, East Qingchun Road 3, Hangzhou, 310016 People’s Republic of China; 2 Department of Cardiology, Key Laboratory of Cardiovascular Intervention and Regenerative Medicine of Zhejiang Province, Sir Run Run Shaw Hospital, Zhejiang University School of Medicine, East Qingchun Road 3, Hangzhou, 310016 People’s Republic of China

**Keywords:** Atrial fibrillation, Catheter ablation, Hemodynamics, Very early period, Echocardiography, Cardiology, Medical research

## Abstract

All chambers’ systolic functions were improved with long-term follow-up after catheter ablation (CA). However, changes on cardiac function on very early phase (within 48 h) has not been thoroughly investigated. This study aimed to evaluate the acute effects of CA on cardiac function and morphology immediately post-procedural (≤ 48 h). We prospectively enrolled 117 consecutive patients. Hemodynamic parameters including biatrial pressure and echocardiographic data were acquired pre- and post-ablation. (i) All bi-atrial pressures significantly elevated post-ablation (p < 0.002). (ii) Bi-atrial function including ejection fraction, reservoir strain and conduit strain improved immediately after ablation (p < 0.01). Persistent atrial fibrillation (AF) improved more than paroxysmal AF. (iii) Biventricular systolic function improved early but left ventricular diastolic function was stunned within 48 h (p < 0.01). Whereas all chambers’ volume did not change significantly (p > 0.05). (iv) No statistically significant differences in pre- versus post-procedural pressure changed between patients with persistent AF and those with paroxysmal AF in biatrial pressure elevation (p > 0.05). (v) Echocardiographic parameters including LA reservoir strain, RA reservoir strain, peak A of mitral valve, peak E of tricuspid valve and inferior vena cava diameters increased more in persistent AF groups (p < 0.05). This prospective study suggested all chambers’ systolic functions improved in early period post-ablation with left ventricular diastolic function stunning. Mechanical improvements preceding structural remodeling in AF patients early after ablation.

**Supplementary Information:**

The online version contains supplementary material available at 10.1038/s41598-025-28591-5.

## Introduction

Atrial fibrillation (AF) is the most prevalent cardiac arrhythmia and is associated with significantly increased mortality and morbidity^1^. AF causes cardiac dysfunction including biatrial systolic function^2,3^, biventricular systolic function and diastolic function^4–6^. Catheter ablation (CA) has emerged as a guideline-recommended treatment for symptomatic AF refractory to antiarrhythmic drug therapy^7,8^.

CA facilitates reverse left atrial (LA) remodeling and improves LA function in AF patients in long-term follow-up^9,10^. Furthermore, CA significantly reduces overall mortality rates and decreases hospitalization for worsening heart failure by improving left ventricular (LV) ejection fraction (EF)^11,12^ and LV diastolic function^4^. Post-ablation period can be divided into acute, subacute and chronic phases. In the acute phase, edema and necrosis occur at the ablation site, while in the chronic phase, fibrosis with scar formation develops at the ablation site^13,14^. However, most studies on cardiac remodeling have focused on phase changes (more than 1 month) in the LA^2,15–17^ and LV^15,18,19^. Data on the acute phase (within 48 h) involving the atrial pressures and the function of LA, LV, right atrium (RA), and right ventricle (RV) remain limited. The impact of ablation on biventricular and biatrial function during the immediate postoperative period has not been thoroughly investigated.

This prospective study aimed to evaluate the acute effects of CA on cardiac function and morphology across all chambers in patients with AF during the immediate post-procedural period (≤ 48 h). We consecutively enrolled 117 AF patients from a tertiary referral center. Hemodynamic assessments included direct measurements of left atrial pressure (LAP) and right atrial pressure (RAP). Comprehensive echocardiographic evaluation of all cardiac chambers’ dimensions and functions was performed at baseline and within 48 h post-ablation. Biatrial and biventricular functions were systematically analyzed using standardized protocols.

## Methods

### Study population

**Fig. 1:**
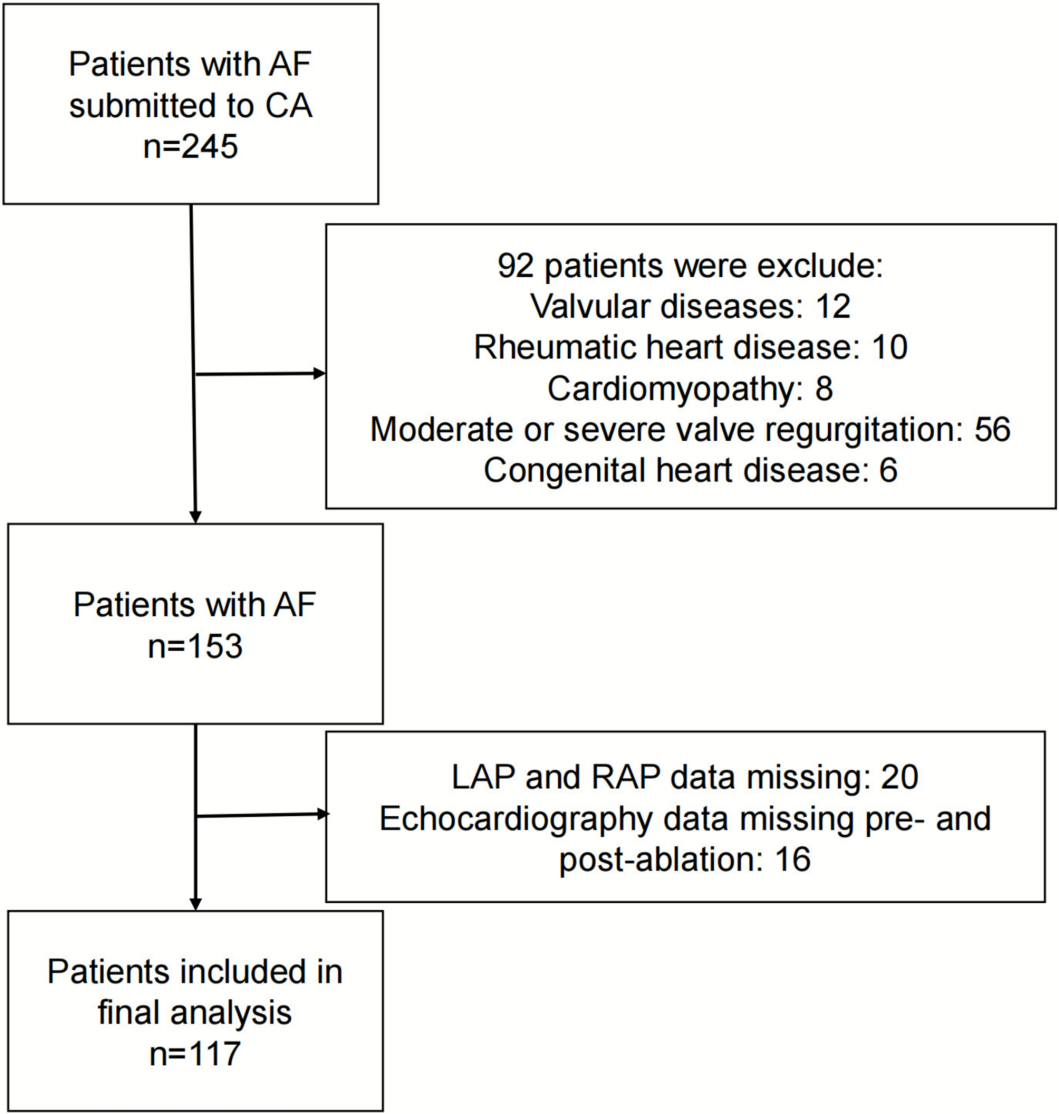
The flowchart of patient selection with atrial fibrillation. AF: atrial fibrillation; CA: catheter ablation; LAP: left atrial pressure; RAP: right atrial pressure.

We prospectively enrolled consecutive patients with persistent or paroxysmal AF undergoing CA at Sir Run Run Shaw Hospital (a tertiary referral center) between February 2024 and February 2025. Paroxysmal AF is characterized by termination within 7 days of onset, either spontaneously or with intervention. Persistent AF is characterized by continuous arrhythmia that lasts for more than 7 days^20^. Among 245 ablation procedures performed during this period, patients were included if they: (1) were ≥ 18 years old, (2) had documented AF, and (3) achieved immediate post-procedural sinus rhythm through radiofrequency ablation. Key exclusion criteria comprised: (1) valvular diseases (mitral valve (MV) prolapse, rheumatic heart disease); (2) congenital heart disease; (3) pericardial disorders (constrictive pericarditis); (4) cardiomyopathy (hypertrophic cardiomyopathy, restrictive cardiomyopathy, etc.); (5) moderate or severe valve regurgitation (significant MV regurgitation, aortic regurgitation and tricuspid regurgitation); (6) Systemic comorbidities (malignancy, connective tissue diseases). After screening based on the exclusion criteria, 117 patients (214 examinations) were ultimately enrolled in the study (Fig. 1). The Institutional Review Board of Sir Run Run Shaw Hospital approved the study protocol (Approval No. 2024-2517-01), which complied with Declaration of Helsinki principles. All participants provided written informed consent prior to enrollment.

### CA

The ablation protocol typically involved pulmonary vein isolation to achieve electrical isolation. An intracardiac echocardiography catheter and sheaths were introduced via the femoral vein. Transseptal puncture was performed under intracardiac echocardiography guidance. The procedure utilized the Carto Sound three-dimensional mapping system (CARTO, Biosense Webster Inc.) with a 3.5 mm irrigated-tip catheter, equipped with contact-force sensing technology (Smart-Touch™, Biosense Webster Inc., Diamond Bar, CA, USA). Additional ablation, including linear ablation, substrate modification and superior vena cava isolation was performed at the operator’s discretion. The ablation settings were as follows: power 30-45W and irrigation flow rate 8–30 ml/min.

### Measurement of RAP and LAP

The RAP and LAP were measured in all patients under stable fluid-loading conditions during CA. No new medications were administered during CA and echocardiography. RAP was measured after access via the femoral vein. LAP was measured immediately after transseptal puncture. Following successful restoration of sinus rhythm by CA, all patients underwent repeat atrial pressure measurement. Physicians used the same catheter system as that used pre-ablation to measure LAP/RAP post-ablation. They measured LAP/RAP parameters pre- and post-ablation included maximum LAP/RAP, mean LAP/RAP, and minimum LAP/RAP. LAP and RAP measurements were taken at end-expiration and represent the mean of ≥ 3 beats (Supplementary data online, Figure S1). The physician confirmed stable waveforms before recording.

### Echocardiography

All participants underwent comprehensive transthoracic echocardiography using a Vivid E95 ultrasound system (GE Vingmed Ultrasound AS, Horten, Norway) equipped with an M5Sc transducer (1.4–4.6 MHz). Standardized examinations were performed: (1) within 1 week before CA, and (2) within 48 h post-procedure, following contemporary guidelines from the European Association of Cardiovascular Imaging and American Society of Echocardiography^21,22^. Thirty patients with persistent AF were in AF rhythm during pre-ablation echocardiographic assessment while nineteen patients were in sinus rhythm. Paroxysmal AF patients were in sinus rhythm during pre-ablation echocardiographic assessment. LV EF was obtained from parasternal long-axis views. Mitral and tricuspid inflow velocities were assessed by pulsed-wave Doppler (sample volume 1–2 mm at mitral or tricuspid leaflet tips) across 5 cardiac cycles in apical 4-chamber view. The diameter of the inferior vena cava (IVC) was measured perpendicular to its long axis, approximately 1–2 cm distal to the RA junction. Assessment of size and collapsibility percentage was performed during quiet respiration^21^. Apical two-chamber, three-chamber and four-chamber views were acquired for five consecutive cycles with frame rates exceeding 60 frames/s and stored for offline analysis later. LV global longitudinal strain (GLS) and LA strain were measured from LV-focused apical views (Supplementary data online, Figure S2). RV dimensions, RA strain and RV strain were measured from a RV-focused apical four-chamber view and two-chamber view according to the current recommendations (Supplementary data online, Figure S3)^22,23^.

### Echocardiographic data analysis

Echocardiographic data were analysed offline by the same operator using proprietary software (version 204, EchoPAC, GE Vingmed Ultrasound, Norway). Measurements for all parameters from three (sinus rhythm) or five (AF) cardiac cycles were averaged. All Doppler measurements were performed at sweep speeds of 50–100 mm/s. LA, RA, LV and RV strains were analysed from measurement list in EchoPAC software according to current recommendations^24^. Timing of valvular events was measured from spectral Doppler recordings. The electrocardiogram R-wave trigger served as the zero strain reference. Endocardial border tracking was verified manually for all strain analyses.

### Statistical analysis

All continuous data were presented as median (interquartile range) for non-normally distributed variables and mean ± standard deviation for normally distributed variables. All categorical data were expressed as numbers and percentages. The preprocedural and postprocedural hemodynamic parameters were analyzed using paired-sample t-tests. Comparisons between AF groups were performed using the independent t-test or Mann–Whitney U test as appropriate. Missing data were imputed using multiple imputation methods. All statistical analyses were performed using the SPSS package 27.0 (SPSS, Inc., Chicago, IL, USA). All statistical graphs were created using GraphPad Prism software (version 10; GraphPad Software, San Diego, CA). Composite figures were subsequently assembled using Adobe Illustrator (version 2023; Adobe Systems). Statistical significance was defined as p < 0.05. The study was exploratory across many parameters and p < 0.05 was applied uniformly without formal correction for multiple comparisons.

## Results

### Study population

Among the 245 participants with AF undergoing CA, we excluded individuals with valvular diseases (n = 12), rheumatic heart disease (n = 10), cardiomyopathy (n = 8), moderate or severe valvular regurgitation (n = 56), congenital heart disease (n = 6), missing LAP and RAP data (n = 20), and missing pre- and post-ablation echocardiography data (n = 16). Finally, 117 patients (214 examinations) were included in the analysis (Fig. 1). Patients’ baseline characteristics and medications were shown in Table 1. Seventy-seven (65.8%) patients were male. Sixty-eight (58.1%) had paroxysmal AF and 49 (41.9%) had persistent AF. The mean age was 65.08 ± 10.14 years. Eleven (9.4%) patients had a history of stroke.

**Table 1:** Baseline characteristics before catheter ablation.

Variables	Value (n = 117)	Paroxysmal AF (n = 68)	Persisitent AF (n = 49)	p value
Demographic and co-morbidities				
Age (year)	65.08 ± 10.14	64.01 ± 10.85	66.55 ± 8.96	0.183
Sex, male, n(%)	77 (65.8%)	44 (64.7%)	33 (67.3%)	0.766
Body surface area, (m^2^)	1.77 ± 0.16	1.78 ± 0.15	1.76 ± 0.17	0.473
Body mass index, (kg/m^2^)	24.43 ± 3.06	24.61 ± 2.99	24.17 ± 3.16	0.438
Heart rate, (beats/min)	76.34 ± 18.77	72.03 ± 17.73	82.32 ± 18.71	0.003
Systolic blood pressure, (mmHg)	125.36 ± 16.56	125.32 ± 17.78	125.42 ± 14.90	0.976
Diastolic blood pressure, (mmHg)	77.45 ± 10.45	76.10 ± 10.63	79.31 ± 10.0	0.102
Cardiac function classification	1 (1–2)	1 (1–2)	1 (1–2)	0.978
CHA2DS2-VASc score	2 (1–3)	2 (1–3)	2 (1–3)	0.880
Diabetes, n (%)	12 (10.3%)	8 (11.8%)	4 (8.2%)	0.526
Hyperlipidemia, n (%)	47 (40.2%)	28 (41.2%)	19 (38.8%)	0.794
Coronary artery disease, n (%)	8 (6.8%)	5 (7.4%)	3 (6.1%)	0.999
Hypertension, n (%)	61 (52.1%)	38(55.9%)	23 (46.9%)	0.339
Chronic kidney disease, n (%)	3 (2.6%)	2(2.9%)	1 (2%)	0.999
Redo, n (%)	24 (20.5%)	15 (22.1%)	9 (18.4%)	0.626
Smoke, n (%)	19 (16.2%)	15 (22.1%)	4 (8.2%)	0.045
Drinking, n (%)	18 (15.9%)	12 (17.6%)	6 (12.2%)	0.424
Prior stroke/TIA, n (%)	11 (9.4%)	6 (8.8%)	5 (10.2%)	0.999
AF type	117 (100%)	68 (58.1%)	49 (41.9%)	–
Duration of AF atrial fibrillation (month)	12 (2–29)	12 (3–24)	7 (1–36)	0.801
Laboratory findings				
Elevated creatinine, n (%)	5 (4.3%)	2 (2.9%)	3 (6.1)	0.707
Hemoglobin, (g/L)	144.03 ± 15.89	143.71 ± 15.38	144.47 ± 16.73	0.799
Elevated pro-BNP or BNP, n (%)	54 (46.2%)	23 (33.8%)	31 (63.3%)	0.002
Medications				
ACEI/ARB, n (%)	42 (35.9%)	28 (41.2%)	14 (28.6%)	0.161
MRA, n (%)	5 (4.3%)	1 (1.5%)	4 (8.2%)	0.193
Diuretics, n (%)	9 (7.7%)	3 (4.4%)	6 (12.2%)	0.224
SGLT2 inhibitor, n (%)	5 (4.3%)	3 (4.4%)	2 (4.1%)	0.999
Beta-blocker, n (%)	51 (43.6%)	32 (47.1%)	19 (38.8%)	0.373
Dihydropyridine Ca channel blocker, n (%)	24 (20.5%)	11 (16.2%)	13 (26.5%)	0.171
Digoxin, n (%)	4 (3.4%)	1 (1.5%)	3 (6.1%)	0.395
Anticoagulant, n (%)	82 (70.1%)	46 (67.6%)	36 (73.5)	0.497

AF: atrial fibrillation; ACEI: Angiotensin-converting enzyme; ARB: angiotension II receptor blocker; MRA: mineralocorticoid receptor antagonist; SGLT2: sodium-glucose cotransporter-2 inhibitor.

Data are expressed as mean ± SD, number (percentage), or median (interquartile range).

### Both LAP and RAP elevated post ablation

**Fig. 2:**
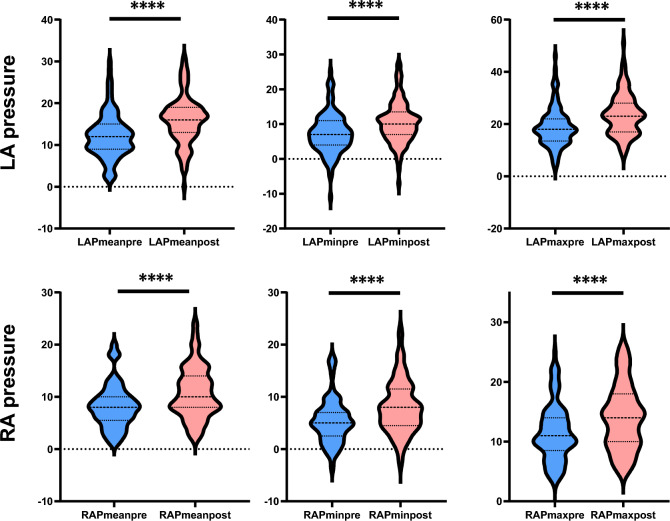
Left atrial pressure and right atrial pressure elevation post-ablation. LA: left atrial; RA: right atrial; max:maximum; min:minimum; pre: pre-ablation; post: post-ablation; ****p < 0.0001.

Among 117 patients, pre-ablation LAP measurements were obtained in 112 patients, while post-ablation LAP measurements were available for 105 patients. Similarly, pre-ablation RAP measurements were acquired in 97 patients and post-ablation RAP measurements in 96 patients. Pre- and post-procedure pressure comparisons were made only among patients with both measurements available. The mean LAP was 12.19 ± 5.35 mmHg pre-ablation and increased significantly to 15.98 ± 5.76 mmHg post-ablation. Similarly, mean RAP increased from 8.19 ± 4.17 mmHg pre-ablation to 10.79 ± 4.87 mmHg post-ablation. changes in LAP and RAP after ablation were shown in Table 2. All LAPs—including maximum, minimum, and mean pressures—demonstrated significant elevation post-ablation compared to pre-ablation values (p < 0.001 for all comparisons). This pattern was mirrored in RAP measurements, with maximum, minimum, and mean pressures significantly increased following the procedure (p < 0.001 for all comparisons) (Fig. 2). No patients experienced clinical symptoms or complications related to the acute pressure or diastolic changes such as acute heart failure, pulmonary edema, or hypotension in the 48 h post-CA.

**Table 2:** LAP and RAP changes after catheter ablation in AF patients.

	Total	Pre-ablation	Post-ablation	p value	POAF pre ablation	Post ablation	p value	PEAF pre ablation	Post ablation	p value
LAP min	8.92 ± 6.12	7.36 ± 5.83	10.49 ± 6.02	< 0.001	6.59 ± 5.54	10.21 ± 5.20	< 0.001	8.42 ± 6.10	10.86 ± 7.04	0.002
LAP max	21.21 ± 8.07	18.77 ± 7.51	23.64 ± 7.89	< 0.001	18.75 ± 7.81	22.96 ± 8.11	< 0.001	18.81 ± 7.16	24.60 ± 7.56	< 0.001
LAP mean	14.08 ± 5.86	12.19 ± 5.35	15.98 ± 5.76	< 0.001	11.75 ± 5.23	15.36 ± 5.46	< 0.001	12.79 ± 5.50	16.84 ± 6.09	< 0.001
RAP min	6.75 ± 4.89	5.42 ± 4.34	8.08 ± 5.06	< 0.001	4.99 ± 3.84	7.60 ± 4.82	< 0.001	6.02 ± 4.93	8.74 ± 5.36	< 0.001
RAP max	12.78 ± 5.23	11.53 ± 4.77	14.03 ± 5.39	< 0.001	11.69 ± 4.75	13.84 ± 5.57	0.001	11.32 ± 4.84	14.30 ± 5.16	< 0.001
RAP mean	9.49 ± 4.71	8.19 ± 4.17	10.79 ± 4.87	< 0.001	7.83 ± 3.79	10.29 ± 4.69	< 0.001	8.71 ± 4.65	11.49 ± 5.07	< 0.001

LAP: left atrial pressure; RAP: righr atrial pressure; AF:atrial fibrillation; POAF: paroxysmal atrial fibrillation; PEAF: persisitent atrial fibrillation.

Data are expressed as mean ± SD.

### Association of AF type in LAP and RAP elevation

We further investigated the impact of AF type on LAP and RAP before and after ablation. No statistically significant differences were observed in LAP elevation between persistent and paroxysmal AF patients. Post-ablation LAP measurements remained comparable between the two AF subtypes. Similarly, for RAP elevation, neither pre- nor post-procedural RAP levels differed significantly between persistent and paroxysmal AF groups (Table 3). The consistent lack of subtype-specific pressure differences suggested the acute post-ablation atrial hemodynamic changes may represent a universal response to procedural injury rather than being influenced by AF sbutype**.**

**Table 3:** Association of AF type in LAP and RAP elevations after ablation.

	Paroxysmal AF	Persitent AF	z or t value	p value
LAP min pre-ablation	6.75 (4.00, 10.00)	7.00 (3.50, 12.00)	1.09	0.276
LAP max pre-ablation	17.95 (13.25, 21.75)	18.00 (13.5, 23.00)	0.498	0.619
LAP mean pre-ablation	12.00 (9.00, 14.75)	12.00 (9.00, 16.50)	0.866	0.386
LAP min post-ablation	11.00 (6.25, 14.00)	9.14 (7.00, 12.24)	− 0.379	0.705
LAP max post-ablation	23.00 (17.05, 25.75)	24.00 (17.50, 30.00)	1.36	0.174
LAP mean post-ablation	15.36 ± 5.46	16.84 ± 6.09	− 1.38	0.170
RAP min pre-ablation	5.00 (2.25, 7.00)	5.00 (2.56, 8.00)	0.712	0.477
RAP max pre-ablation	11.00 (9.00, 14.60)	10.64 (7.50, 14.23)	− 0.390	0.696
RAP mean pre-ablation	8.00 (5.00, 10.01)	8.09 (6.00, 10.00	0.891	0.373
RAP min post-ablation	7.00 (4.00, 11.00)	8.00 (5.07, 11.97)	0.932	0.351
RAP max post-ablation	13.72 (9.00, 18.00)	14.12 (10.13, 16.98)	0.437	0.662
RAP mean post-ablation	9.75 (7.00, 13.73)	11.00 (8.00, 15.00)	1.068	0.286
ΔLAP min	3.00 (1.00, 5.75)	2.00 (− 1.00, 4.50)	− 1.785	0.074
ΔLAP max	4.00 (0.00, 8.00)	6.00 (1.00, 9.50)	1.295	0.195
ΔLAP mean	3.00 (0.25, 7.00)	3.00 (1.00, 7.00)	0.222	0.825
ΔRAP min	2.00 (0.00, 4.00)	3.00 (0.00, 4.50)	0.533	0.594
ΔRAP max	2.00 (− 0.75, 5.00)	2.00 (1.00, 6.00)	1.069	0.285
ΔRAP mean	2.00 (0.00, 4.75)	3.00 (1.00, 5.00)	0.745	0.456

AF: atrial fibrillation; LAP: left atial pressure; RAP: right atrial pressure; Δ: pressure post-ablation minus pre-ablation.

### All cardiac chambers’ systolic functions began to recover within 48 h after CA

**Fig. 3:**
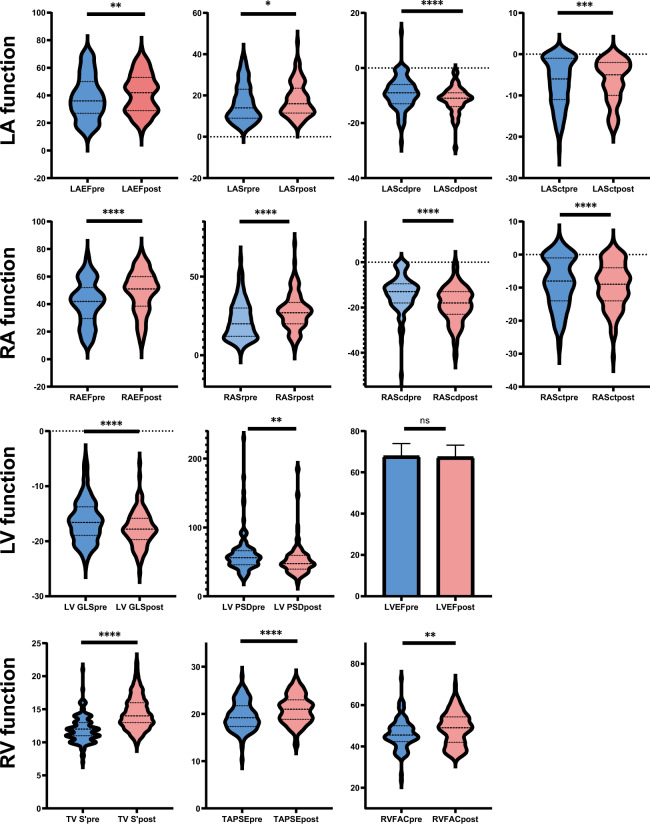
Functional changes of all cardiac chambers after ablation within 48 h. LA: left atrial; RA: right atrial; LAEF: left atrial ejection fraction; LASr: left atrial reservoir strain; LAScd: left atrial conduit strain; LASct: left atrial contractile strain; RAEF: right atrial ejection fraction; RASr: right atrial reservoir strain; RAScd: right atrial conduit strain; RASct: right atrial contractile strain; LV GLS: left ventricular global longitudinal strain; PSD: peak strain dispersion; LV EF: left ventricular ejection fraction; TV S’: tricuspid annular peak systolic velocity; TAPSE: tricuspid annular plane systolic excursion; RVFAC: right ventricular fractional area change; pre: pre-ablation; post: post-ablation; *p < 0.05; **p < 0.01; ***p < 0.001; ****p < 0.0001; ns: not significant.

All cardiac chambers’ systolic functions began to recover in the very early postoperative period after AF ablation (Table 4). Data suggested significant improvement in LA reservoir strain (Sr), conduit strain (Scd) (p < 0.05), with particularly notable reduction in LA contractile strain (Sct). Atrial function improved more in the persistent AF group than in the paroxysmal AF group. Given that 61.2% of patients with persistent AF were in AF rhythm during baseline echocardiography, conversion from AF to sinus rhythm could account for part of the immediate functional improvement. However, no significant improvement was observed in LA morphological parameters (p > 0.05). Both LV GLS (from − 16.34 to − 17.72%, p < 0.001) and peak strain dispersion (from 60.84 to 53.3 ms, p = 0.007) showed marked improvement compared to preoperative values, indicating recovery of LV systolic function and synchrony post-procedure, though no statistically significant difference was found in LV EF, and LV structural parameters remained unchanged. RA function parameters RA Sr and RA Scd exhibited significant postoperative recovery, while RA Sct showed no significant alteration. RV functional parameters showed statistically significant improvement, including TV S’ peak velocity, TAPSE and RVFAC. (Fig. 3) While RV GLS and free wall strain exhibited a numerical trend toward improvement postoperatively (RV GLS: − 16.32% vs. − 17.03%; FWS: − 18.53% vs. − 18.82%), these changes did not reach statistical significance (p = 0.227 and p = 0.768, respectively). Similarly, neither RA nor RV structural parameters showed significant changes. Functional improvements were more pronounced in persistent AF patients than in paroxysmal AF patients across all chambers.

**Table 4:** Post-procedural echcardiography findings within 48 h after catheter ablation.

	Total (n = 117)	Pre-ablation	Post-ablation	p value	POAF pre-ablation	post-ablation	p value	PEAF pre-ablation	post-ablation	p value
BP and heart rate										
Systolic BP (mmHg)	131.89 ± 19.59	133.59 ± 20.05	130.19 ± 19.04	0.065	131.76 ± 20.91	132.35 ± 20.03	0.800	136.13 ± 18.71	127.19 ± 17.33	0.002
Diastolic BP (mmHg)	83.35 ± 13.03	83.89 ± 13.70	82.80 ± 12.36	0.398	80.39 ± 11.40	81.99 ± 12.28	0.269	88.75 ± 15.18	83.93 ± 12.51	0.035
Heart rate (beats/min)	76.82 ± 14.88	78.01 ± 16.98	75.64 ± 12.39	0.118	77.08 ± 20.11	74.07 ± 12.59	0.149	79.31 ± 11.37	77.82 ± 11.88	0.500
LA structural parameters										
LAAPD (mm)	39.48 ± 5.36	39.11 ± 5.61	39.84 ± 5.10	0.081	37.01 ± 5.10	37.98 ± 4.47	0.060	42.03 ± 4.99	42.42 ± 4.82	0.573
LA max (ml)	61.16 ± 21.52	60.47 ± 21.41	61.84 ± 21.70	0.339	54.67 ± 19.70	54.50 ± 19.69	0.920	68.52 ± 21.27	72.03 ± 20.36	0.168
LA min (ml)	38.34 ± 19.61	38.87 ± 20.13	37.81 ± 19.15	0.423	31.36 ± 16.86	30.27 ± 16.88	0.471	49.31 ± 19.79	48.28 ± 17.21	0.669
LA PreA (ml)	47.89 ± 20.16	48.61 ± 20.83	47.16 ± 19.53	0.343	42.16 ± 17.96	40.57 ± 17.87	0.360	57.57 ± 21.38	56.32 ± 18.14	0.651
LA volume (ml)	62.49 ± 22.71	62.63 ± 23.37	62.35 ± 22.12	0.864	56.22 ± 21.08	55.71 ± 20.61	0.781	71.53 ± 23.69	71.56 ± 20.99	0.993
LA volume index(ml/m2)	35.23 ± 12.84	35.38 ± 13.45	35.09 ± 12.26	0.764	31.12 ± 12.17	31.21 ± 11.29	0.928	41.29 ± 13.00	40.48 ± 11.59	0.657
LA stiffness	0.83 (0.55, 1.36)	0.78 (0.46, 1.22)	0.95 (0.60, 1.45)	0.345	0.57 (0.35, 0.88)	0.72 (0.48, 1.13)	0.282	1.00 (0.79, 1.63)	1.33 (0.84, 1.97)	0.732
LA function										
LA EF (%)	40.13 ± 15.10	38.71 ± 15.94	41.55 ± 14.13	0.012	45.53 ± 15.78	47.17 ± 13.92	0.295	29.23 ± 10.44	33.74 ± 10.26	0.006
LA reservior strain (%)	17.22 ± 8.60	16.49 ± 9.01	17.95 ± 8.14	0.018	20.35 ± 9.24	21.10 ± 8.70	0.418	11.13 ± 5.16	13.57 ± 4.55	0.001
LA conduit strain (%)	− 10.33 ± 5.34	− 9.22 ± 5.70	− 11.43 ± 4.73	< 0.0001	− 10.23 ± 6.44	− 12.59 ± 5.34	0.005	− 7.82 ± 4.13	− 9.82 ± 3.11	0.007
LA contractile strain (%)	− 6.66 ± 5.48	− 8.52 ± 7.56	− 6.45 ± 5.13	0.001	− 9.39 ± 5.70	− 8.38 ± 5.21	0.092	− 3.40 ± 3.94	− 3.77 ± 3.60	0.526
RA structural parameters										
RA max (ml)	41.44 ± 17.64	40.87 ± 16.62	42.00 ± 18.66	0.482	38.16 ± 14.70	38.72 ± 20.51	0.783	44.63 ± 18.47	46.56 ± 14.77	0.470
RA min (ml)	23.68 ± 14.62	25.29 ± 14.86	22.06 ± 14.25	0.004	21.21 ± 12.80	17.99 ± 14.33	0.013	30.97 ± 15.75	27.70 ± 12.18	0.109
RA preA (ml)	32.10 ± 14.72	33.16 ± 14.40	31.04 ± 15.01	0.134	29.85 ± 11.45	27.44 ± 15.30	0.154	37.74 ± 16.77	36.04 ± 13.20	0.487
RA area (cm2)	17.02 ± 4.61	16.68 ± 4.32	17.35 ± 4.87	0.098	15.69 ± 4.06	16.14 ± 4.77	0.348	18.05 ± 4.35	19.03 ± 4.53	0.168
RA volume index (ml/m^2^)	23.25 ± 10.11	22.80 ± 9.81	23.70 ± 10.42	0.323	20.74 ± 8.15	21.67 ± 11.28	0.413	25.65 ± 11.22	26.53 ± 8.42	0.571
RA function										
RA reservior strain (%)	25.15 ± 12.08	22.33 ± 12.15	27.96 ± 11.38	< 0.0001	27.66 ± 12.47	30.97 ± 12.02	0.060	14.95 ± 6.62	23.79 ± 8.98	< 0.0001
RA conduit strain (%)	− 16.16 ± 8.25	− 13.66 ± 7.84	− 18.66 ± 7.92	< 0.0001	− 15.62 ± 8.68	− 19.85 ± 8.75	0.001	− 10.94 ± 5.51	− 17.00 ± 6.31	< 0.0001
RA contractile strain (%)	− 8.89 ± 7.18	− 8.52 ± 7.56	− 9.26 ± 6.79	0.324	11.88 ± 7.10	− 11.18 ± 6.92	0.500	− 3.85 ± 5.42	− 6.60 ± 5.67	0.008
RA EF (%)	45.19 ± 16.00	41.24 ± 15.81	49.13 ± 15.26	< 0.0001	47.21 ± 14.65	54.59 ± 13.44	< 0.0001	32.96 ± 13.57	41.56 ± 14.50	0.001
LV structural parameters										
IVSd (mm)	9.63 ± 1.47	9.55 ± 1.49	9.70 ± 1.45	0.317	9.59 ± 1.47	9.93 ± 1.44	0.080	9.51 ± 1.53	9.39 ± 1.43	0.633
LVIDd (mm)	48.17 ± 4.28	47.94 ± 4.44	48.41 ± 4.12	0.284	47.28 ± 4.69	47.38 ± 4.04	0.877	48.84 ± 3.94	49.84 ± 3.84	0.119
LVPWd (mm)	9.34 ± 1.25	9.36 ± 1.28	9.32 ± 1.23	0.780	9.23 ± 1.22	9.35 ± 1.21	0.505	9.54 ± 1.35	9.28 ± 1.27	0.266
End-diastolic volume (ml)	110.01 ± 21.54	108.32 ± 21.70	111.71 ± 21.35	0.119	104.65 ± 22.29	105.72 ± 20.39	0.709	113.41 ± 19.96	120.02 ± 19.99	0.049
End-systolic volume (ml)	36.24 ± 10.98	35.39 ± 10.80	37.09 ± 11.13	0.096	32.91 ± 10.07	33.95 ± 8.95	0.411	38.83 ± 10.94	41.45 ± 12.41	0.126
Relative wall thickness	0.38 ± 0.06	0.38 ± 0.07	0.38 ± 0.05	0.896	0.39 ± 0.08	0.39 ± 0.05	0.292	0.37 ± 0.07	0.37 ± 0.05	0.610
LV systolic function										
LV EF (%)	67.53 ± 6.04	67.74 ± 6.22	67.33 ± 5.86	0.530	69.12 ± 5.44	67.94 ± 4.91	0.161	65.83 ± 6.78	66.48 ± 6.94	0.542
Fractional shortening (%)	37.73 ± 4.57	37.85 ± 4.74	37.62 ± 4.41	0.667	38.83 ± 4.32	38.03 ± 3.99	0.245	36.49 ± 4.99	37.05 ± 4.93	0.515
LV GLS (%)	− 17.03 ± 3.61	− 16.34 ± 3.86	− 17.72 ± 3.20	< 0.0001	− 17.73 ± 3.12	− 18.62 ± 3.10	0.032	− 14.41 ± 3.99	− 16.46 ± 2.94	< 0.0001
Peak systolic delay (ms)	57.07 ± 27.01	60.84 ± 27.91	53.30 ± 25.65	0.007	58.32 ± 24.80	53.44 ± 29.24	0.154	64.34 ± 31.66	53.11 ± 19.92	0.018
LV diastolic function										
MV E (cm/s)	85.33 ± 19.29	81.49 ± 19.51	89.18 ± 18.36	< 0.0001	75.70 ± 14.84	86.50 ± 18.75	< 0.0001	89.53 ± 22.34	92.89 ± 17.32	0.232
E wave acceleration (m/s2)	16.04 ± 13.45	16.69 ± 16.89	15.39 ± 8.79	0.436	12.57 ± 5.37	14.79 ± 8.54	0.046	22.40 ± 24.33	16.21 ± 9.16	0.091
E wave deceleration time (ms)	167.77 ± 60.75	169.69 ± 64.62	165.85 ± 56.83	0.522	185.86 ± 71.88	169.88 ± 61.82	0.064	147.24 ± 44.66	160.28 ± 49.15	0.085
MV A (cm/s)	–	–	–	–	63.64 ± 20.57	59.73 ± 19.98	0.080	–	–	–
MV E/A	–	–	–	–	1.34 ± 0.53	1.68 ± 0.86	0.003	–	–	–
MED E’ (cm/s)	8.20 ± 1.72	8.34 ± 1.95	8.06 ± 1.45	0.120	8.14 ± 1.88	8.12 ± 1.39	0.920	8.60 ± 2.04	7.97 ± 1.55	0.038
LAT E’ (cm/s)	10.43 ± 2.54	11.44 ± 2.40	9.43 ± 2.27	< 0.0001	11.27 ± 2.38	9.52 ± 2.22	< 0.0001	11.67 ± 2.43	9.30 ± 2.36	< 0.0001
MED E/E'	10.79 ± 3.05	10.17 ± 3.02	11.41 ± 2.96	< 0.0001	9.72 ± 2.86	10.93 ± 2.88	0.001	10.80 ± 3.15	12.07 ± 2.97	0.004
LAT E/E'	8.63 ± 2.76	7.43 ± 2.30	9.83 ± 2.67	< 0.0001	7.05 ± 2.01	9.44 ± 2.77	< 0.0001	7.96 ± 2.57	10.35 ± 2.45	< 0.0001
Mean E/E'	9.51 ± 2.68	8.58 ± 2.52	10.44 ± 2.52	< 0.0001	8.16 ± 2.24	10.03 ± 2.58	< 0.0001	9.16 ± 2.78	11.00 ± 2.34	< 0.0001
MV Vp (cm/s)	70.81 ± 27.97	68.67 ± 29.27	72.94 ± 26.56	0.149	66.56 ± 23.27	71.82 ± 23.92	0.165	71.60 ± 36.04	74.51 ± 30.03	0.546
E/Vp	1.44 ± 0.71	1.39 ± 0.75	1.48 ± 0.66	0.209	1.35 ± 0.76	1.42 ± 0.63	0.522	1.45 ± 0.75	1.56 ± 0.71	0.201
PV S wave (cm/s)	58.45 ± 16.25	53.22 ± 13.34	63.67 ± 17.25	< 0.0001	54.99 ± 14.53	63.01 ± 17.56	0.003	50.78 ± 11.18	64.59 ± 16.93	< 0.0001
PV D wave (cm/s)	48.82 ± 16.48	45.78 ± 15.76	51.85 ± 16.70	0.002	46.45 ± 14.56	54.29 ± 17.56	0.001	44.86 ± 17.39	48.47 ± 14.95	0.265
PV atrial reversal wave (cm/s)	30.97 ± 7.68	30.43 ± 7.34	31.52 ± 8.00	0.236	30.61 ± 8.07	32.45 ± 8.33	0.174	30.17 ± 6.27	30.23 ± 7.40	0.961
PV atrial reversal duration (ms)	164.70 ± 43.27	163.52 ± 39.25	165.88 ± 47.08	0.658	156.63 ± 33.54	163.82 ± 47.93	0.292	173.09 ± 44.64	168.74 ± 46.22	0.614
PV S/D	1.32 ± 0.51	1.29 ± 0.50	1.35 ± 0.52	0.332	1.27 ± 0.50	1.25 ± 0.48	0.722	1.32 ± 0.49	1.49 ± 0.54	0.076
PV D duration (ms)	132.90 ± 50.93	137.31 ± 50.57	128.50 ± 51.12	0.190	134.17 ± 46.92	133.29 ± 54.44	0.919	141.66 ± 55.45	121.85 ± 45.84	0.065
IVRT (ms)	73.31 ± 18.46	75.64 ± 17.98	71.00 ± 18.72	0.003	77.00 ± 18.30	71.91 ± 19.88	0.021	73.74 ± 17.53	69.71 ± 17.10	0.055
Peak TR velocity (m/s)	2.67 ± 0.36	2.59 ± 0.37	2.74 ± 0.33	< 0.0001	2.58 ± 0.37	2.67 ± 0.32	0.086	2.60 ± 0.37	2.84 ± 0.34	< 0.0001
RV Systolic Pressure (mmHg)	34.30 ± 7.81	32.22 ± 6.98	36.39 ± 8.05	< 0.0001	31.34 ± 6.78	34.75 ± 7.18	0.001	33.44 ± 7.14	38.67 ± 8.70	< 0.0001
RV systolic function										
TV S’ peak (cm/s)	13.20 ± 2.60	11.92 ± 2.03	14.48 ± 2.48	< 0.0001	12.17 ± 1.95	15.10 ± 2.54	< 0.0001	11.57 ± 2.11	13.62 ± 2.14	< 0.0001
TAPSE (mm)	20.27 ± 3.10	19.58 ± 3.05	20.96 ± 3.00	< 0.0001	20.32 ± 3.17	21.17 ± 3.14	0.100	18.54 ± 2.58	20.67 ± 2.79	< 0.0001
RV fractional area change (%)	47.36 ± 7.61	46.04 ± 7.19	48.68 ± 7.82	0.005	47.41 ± 7.45	48.88 ± 7.25	0.189	44.12 ± 6.40	48.40 ± 8.61	0.008
RV GLS (%)	− 16.67 ± 5.20	− 16.32 ± 5.06	− 17.03 ± 5.34	0.227	'− 17.81 ± 4.76	− 17.69 ± 5.49	0.872	'− 14.25 ± 4.78	'− 16.11 ± 5.03	0.059
Free wall strain (%)	− 18.67 ± 7.94	− 18.53 ± 6.59	− 18.82 ± 9.12	0.768	− 20.24 ± 5.92	− 19.08 ± 9.96	0.391	− 16.15 ± 6.79	− 18.46 ± 7.90	0.117
RV diastolic function										
TV E peak (cm/s)	57.06 ± 17.56	54.31 ± 15.92	59.81 ± 18.71	0.006	52.50 ± 14.38	61.78 ± 20.23	< 0.0001	56.83 ± 17.69	57.08 ± 16.19	0.933
TV A peak (cm/s)	–	–	–	–	42.65 ± 13.48	49.88 ± 19.25	0.007	–	–	–
TV E/A	–	–	–	–	1.36 ± 0.48	1.30 ± 0.34	0.358	–	–	–
TV E’ (cm/s)	12.42 ± 3.24	12.03 ± 3.27	12.82 ± 3.18	0.045	10.99 ± 2.79	12.35 ± 2.94	0.005	13.48 ± 3.35	13.46 ± 3.41	0.977
TV E/E'	4.86 ± 1.94	4.81 ± 2.03	4.91 ± 1.84	0.581	5.09 ± 2.33	5.25 ± 1.94	0.558	4.42 ± 1.48	4.44 ± 1.59	0.921
IVC max (mm)	13.19 ± 4.09	12.21 ± 3.79	14.16 ± 4.16	< 0.0001	12.14 ± 3.89	13.13 ± 4.10	0.069	12.31 ± 3.70	15.59 ± 3.85	< 0.0001
IVC min (mm)	5.79 ± 3.14	4.85 ± 2.21	6.74 ± 3.63	< 0.0001	4.93 ± 2.49	6.10 ± 3.59	0.006	4.73 ± 1.76	7.63 ± 3.54	< 0.0001
IVC-collapsibility index (%)	57.45 ± 10.84	60.47 ± 7.07	54.43 ± 12.95	< 0.0001	60.31 ± 7.38	56.00 ± 11.42	0.005	60.69 ± 6.68	52.24 ± 14.66	0.001

POAF: paroxysmal atrial fibrillation; PEAF: persisitent atrial fibrillation; BP: blood pressure; LA: left atrial; LAAPD: left atrial anteroposterior diamete; EF: ejection fraction; RA: right atrial; LV: left ventricular; GLS: global longitudinal strain; MV: mitral valve; E:early filling; A: late filling; E': early diastolic; VP: velocity of propagation; PV: pulmonary vein; IVRT: isovolumic relaxation time; TR: tricuspid regurgitation; RV: right ventricular; TV: tricuspid valve; TAPSE: tricuspid annular plane systolic excursion; IVC: inferior vena cava.

Data are expressed as mean ± SD.

Notably, LV diastolic function parameters E/A, E/Eʹ, isovolumic relaxation time, peak tricuspid regurgitation velocity, pulmonary vein S wave and D wave were significantly different postoperatively (p < 0.05). LV filling pressure indices (E/A, E/Eʹ) increased, relaxation parameters (isovolumic relaxation time) shorten, peak tricuspid regurgitation velocity was higher, consistent with a temporary worsening of diastolic function, even as systolic function improved. As 61.2% patients with persistent AF were in AF rhythm during pre-ablation echocardiographic assessment, the A peak was absent on echocardiography in these patients. Therefore, E/A could not be calculated in the persistent AF group. Similarly, RV diastolic function parameters, including tricuspid valve (TV) E peak velocity, TV E’ on tissue Dopple imaging, IVC diameter and IVC collapsibility index, were significantly different postoperatively compared to preoperative values.

### Association of AF type with echocardiographic parameters’ changes

Further analysis of the magnitude of pre- and postoperative changes in relation to AF type revealed that most parameters showed no significant association with AF subtype. However, a few indicators—including RA Sr, MV A veloctiy, TV E velocity, and IVC diameter—demonstrated significant differences. Patients with persistent AF exhibited greater post-operative changes in these parameters than those with paroxysmal AF. Although the post-operative change in LA stiffness did not reach statistical significance between the two AF types, both pre- and post-operative LA stiffness values were higher in persistent AF patients compared to paroxysmal AF patients. This pattern suggests that LA remodeling is more severe in persistent AF than in paroxysmal AF (Table 5).

**Table 5:** Association of AF type in echocardiopgraphic parameters changes after ablation.

	Paroxysmal AF	Persistent AF	z value or t value	p value
LA function				
ΔLA ejection fraction (%)	1.63 ± 12.75	4.51 ± 11.01	− 1.28	0.205
ΔLA reservior strain (%)	1.00 (− 3.93, 4.81)	3.00 (− 1.00, 6.00)	1.98	0.048
ΔLA conduit strain (%)	− 2.36 ± 6.66	− 2.00 ± 4.95	− 0.32	0.749
ΔLA contractile strain (%)	1.00 (− 2.00, 4.75)	− 1.00 (− 3.00, 3.00)	− 1.89	0.059
ΔLA stiffness	0.16 (− 0.07, 0.30)	0.13 (− 0.30, 0.55)	− 0.243	0.808
LA stiffiness pre-ablation	0.57 (0.35, 0.88)	1.00 (0.79, 1.63)	5.05	< 0.001
LA stiffiness post-ablation	0.72 (0.48, 1.13)	1.33 (0.84, 1.97)	4.46	< 0.001
RA function				
ΔRA reservior strain (%)	3.32 ± 14.31	8.84 ± 10.69	− 2.28	0.024
ΔRA conduit strain (%)	− 4.00 (− 11.73, 0)	− 5.00 (− 9.00, − 0.50)	− 0.78	0.436
ΔRA ejection fraction (%)	7.37 ± 15.85	8.60 ± 16.20	− 0.41	0.683
ΔRA min (ml)	− 3.50 (− 9.00, 3.00)	− 4.00 (− 13.00, 4.50)	− 0.19	0.849
LV function				
ΔLV global longitudinal strain (%)	− 0.89 ± 3.34	− 2.05 ± 3.62	1.79	0.076
ΔLV peak systolic delay (ms)	− 8.05 (− 15.84, 4.45)	− 6.74 (− 18.28, 2.65)	− 0.46	0.643
ΔMV E (cm/s)	− 3.00 (− 18.00, 7.23)	− 5.00 (− 18.79, 5.00)	− 1.68	0.094
ΔMV A (cm/s)	− 2.50 (− 17.00, 7.75)	7.0 (− 0.50, 37.5)	4.29	< 0.001
ΔMV E/A	− 0.19 (− 0.15, 0.53)	0.19 (− 0.39, 0.97)	0.20	0.838
ΔLAT E’ (cm/s)	− 2.00 (− 3.53, − 0.15)	− 3.00 (− 4.00, − 1.00)	− 1.38	0.169
ΔMED E/E'	1.22 ± 2.77	1.27 ± 2.92	− 0.10	0.921
ΔLAT E/E'	2.39 ± 2.53	2.39 ± 2.51	− 0.001	0.999
ΔMean E/E'	1.87 ± 2.37	1.85 ± 2.40	0.05	0.959
ΔPV S wave (cm/s)	8.02 ± 21.26	13.81 ± 19.12	− 1.51	0.133
ΔPV D wave (cm/s)	6.83 (− 5.63, 4.45)	4.00 (− 6.00, 14.00)	− 0.75	0.451
ΔIsovolumic relaxation time (ms)	− 5.09 ± 17.78	− 4.03 ± 14.37	− 0.34	0.732
ΔPeak tricuspid regurgitation velocity (m/s)	0.09 (− 0.16, 0.38)	0.25 (0.03, 0.44)	1.81	0.071
ΔRV Systolic Pressure (mmHg)	3.40 ± 7.70	5.23 ± 6.32	− 1.36	0.175
RV function				
ΔTV S’ peak (cm/s)	3.00 (1.00, 4.00)	2.00 (0.04, 3.95)	− 1.81	0.070
ΔTAPSE (mm)	0.85 ± 4.20	2.13 ± 2.86	− 1.96	0.053
ΔRV fractional area change (%)	1.47 ± 9.11	4.28 ± 10.89	− 1.52	0.132
ΔTV E peak (cm/s)	5.78 (− 5.75, 20.00)	− 2.00 (− 12.00, 13.50)	− 2.36	0.018
TV E’ (cm/s)	1.36 ± 3.86	− 0.02 ± 4.51	1.78	0.078
ΔIVC max (mm)	0.99 ± 4.41	3.28 ± 4.29	− 2.82	0.006
ΔIVC min (mm)	0.60 (− 0.69, 1.88)	2.51 (0.05, 4.90)	3.01	0.003
ΔIVC collapsibility index (%)	− 3.36 (− 9.54, 3.04)	− 5.57 (− 15.42, 2.83)	− 1.37	0.171

AF: atrial fibrillation; LA: left atrial; Δ: values post-ablation minus pre-ablation; RA: right atrial; LV: left ventricular; MV: mitral valve; PV: pulmonary vein; RV: right ventricular; TAPSE: tricuspid annular plane systolic excursion; TV: tricuspid valve ; IVC: inferior vena cava. Data are expressed as mean ± SD, or median (interquartile range).

## Discussion

Our study provides novel insights into early hemodynamic changes in AF patients following CA. The key innovation lies in our comprehensive assessment of very early functional and hemodynamic alterations across all cardiac chambers using multimodal echocardiography and bi-atrial pessures. Furthermore, this study suggested transient left ventricular diastolic dysfunction using multiple hemodynamic parameters. These findings expand understanding of acute cardiac recovery patterns post-CA and raise considerations for early post-procedural management, providing insights into how ablation impacts cardiac functions within the critical 48-h window.

### Biatrial pressures elevated immediately after CA

All LAPs (maximum LAP, minimum LAP, and mean LAP) were significantly elevated post-ablation. Elevated LAP is one of the most critical diagnostic features of heart failure with preserved EF. As AF burden increases, particularly in patients with heart failure with preserved EF, there is a progressive decline in LA compliance and hemodynamic efficiency^25,26^. We speculated several factors may contribute to LAP elevation: (1) LV diastolic dysfunction (2) ablation-induced acute inflammation and edema of the atrial wall^27^; (3) LA stunning during the early post-procedural period, evidenced by reduced LASct^28,29^; (4) procedural fluid infusion. During the ablation procedure, the irrigation fluid may lead to increased blood volume, further elevating atrial pressure. This factor could represent one of the limitations in the current study. A fluid volume load of 500–800 mL was administered to each patient during the ablation. However, not all patients’ LA pressure elevated after ablation. In some patients, LA pressure decreased post-ablation. This indicated that volume increase was not the sole factor contributing to atrial pressure elevation. Prior investigations demonstrated that a rapid 500 mL fluid bolus over 8 min could acutely elevate LA pressure by 8.5 mmHg and enlarge its diameter by 4.8 mm^30^. In this study, the volume increase was a relatively gradual process, extending over a period of approximately 1–2 h. No statistically significant differences were observed in LA size and volume. Additionally, the left-to-right shunt caused by transseptal puncture during the ablation procedure can partially offset the LA pressure elevation induced by volume overload. Importantly, volume overload cannot lead to higher E/E’^31^. In this research, even after the volumetric effects resolved by the following day, echocardiographic parameters (including E/E’, peak tricuspid regurgitation velocity and E’ velocity) changed significantly, while LA and RA volume remained unchanged, which suggested persistent LV diastolic dysfunction, indicating that post-ablation LV stunning remains present. Therefore, we concluded that the elevation in LA pressure resulted from the combined effects of LV diastolic dysfunction, impaired LA compliance and volume overload. These changes collectively suggest that the acute elevation in LAP reflects both direct atrial tissue injury and secondary hemodynamic alterations.

Similarly, RAPs increased significantly after CA. Elevated LAP may exacerbate tricuspid regurgitation severity and increase RV systolic pressure, resulting in secondary RAP elevation during the early post-procedural period. Comparative analysis revealed no statistically significant differences in pre- to post-procedural pressure changes between persistent AF and paroxysmal AF patients. This finding indicates that the degree of post-ablation atrial pressure elevation is comparable across AF subtypes, suggesting that the observed pressure increase is primarily attributable to the CA procedure itself and the consequent tissue injury response, rather than to the specific AF classification.

### CA and LA function

Speckle-tracking echocardiography was employed for dynamic assessment of atrial myocardial motion. This technique has emerged as a novel approach for evaluating atrial function due to its angle independence, real-time capabilities, and quantitative measurement of global atrial performance^32,33^. Speckle-tracking based analysis demonstrated a significant post-procedural increase in LA Sr, LA Scd, and LA EF, whereas LA Sct was significantly decreased. The results suggested that the LA’s capacity to store pulmonary venous return during ventricular systole and passive LA emptying during early ventricular diastole was improved significantly. This interpretation aligns with the elevated S- and D-peak velocities of pulmonary venous flow observed in our results^34^. However, active LA contraction during late ventricular diastole was decreased post-procedure. The decrease in LA Sct may be caused by LA stunning after ablation as reported in previous studies reported^35,36^. Our results suggest that such mechanical stunning (especially of the LA) occurs even when sinus rhythm is restored via catheter ablation, consistent with prior observations in post-ablation atrial flutter. Besides, we show that despite this transient setback in atrial contractility, the reservoir and conduit function of the atria and the systolic function of the ventricles improve almost immediately. LA function improved more in persistent AF patients than paroxysmal AF patients. The conversion from AF to sinus rhythm rather than the ablation lesion effects could account for much of the immediate functional improvement. Therefore, the improvement of LA function may be due to restoration of sinus rhythm.

Given that LA stiffness is closely associated with fibrosis^27^, the early phase following CA is characterized by inflammation and edema of the atrial wall, with fibrosis developing only during the later stages as inflammation gradually subsides. Our findings indicate that LA stiffness showed no significant change in the early post-ablation period, which supports this pathophysiological progression. Importantly, patients with persistent AF exhibited significantly higher LA stiffness both preoperatively and postoperatively compared to those with paroxysmal AF (p < 0.001). The correlation between elevated LA stiffness and atrial fibrosis, which serves as a key marker of LA remodeling, suggests that LA structural remodeling is more severe in patients with persistent AF compared to those with paroxysmal AF. This finding is consistent with other research^37–39^.

### CA and RA function

RA systolic function improved significantly within 48 h after CA. RA systolic parameters, including RA Sr, RA Scd and RA EF, were significantly enhanced post-procedure. However, RA Sct showed no significant change. TV A-wave velocity also showed no significant difference after ablation. The absence of significant changes in both RA Sct and TV A velocity may indicate the absence of RA stunning following ablation. In addition, no significant alterations were observed in LA volumes (maximum LAV, LAV index), LA diameter (parasternal long-axis), RA maximum volume, RA volume index or RA area. These results collectively suggested that biatrial systolic function improved early after ablation, preceding structural remodeling.

Compared to patients with paroxysmal AF, those with persistent AF exhibited significantly greater postoperative improvements in LA Sr and RA Sr, accompanied by more substantial reductions in IVC diameter and its respiratory variation index. These findings suggest that persistent AF is associated with more dynamic atrial strain recovery following ablation. However, no statistically significant differences were observed in other atrial functional parameters between the two groups.

### CA and bi-ventricular function

Biventricular systolic function recovered early after CA in AF patients in the present research. The concurrent improvement in LV GLS and reduction in peak strain dispersion suggests early restoration of LV mechanical function and resynchronization during the early post-ablation period. These findings align with long-term follow-up data from prior studies^33,40^. Moreover, the absence of significant change in LV EF underscores the superior sensitivity of speckle-tracking echocardiography over conventional LV EF in detecting subtle, early functional improvements^41,42^. The observed dissociation between functional recovery (↑LVGLS, ↓peak strain dispersion) and static volumetric parameters (LVIDd, EDV) indicates that electromechanical improvements precede geometric remodeling in the early post-ablation period.

Data on RV function changes post-ablation are limited. Kim et al. investigated RV function improved at 1-year follow-up after ablation^43^. However, the exact timing of RV functional recovery has not been reported previously. Our research explored RV function changes within 2 days after ablation. RV systolic parameters, including RVFAC, TAPSE and TV S’ velocity, increased significantly post-procedure.

Minamisaka et al. reported transient LV diastolic dysfuntion, as evaluated by increased E/E’ after ablation. This research assessed LV diastolic function with multiple hemodynamic parameters, including atrial pressures, MV E peak velocity, MV E/A, E/E’, and tricuspid regurgitation velocity. In addition, we found that IVC diameter and its IVC collapsibility index also changed significantly postoperatively. When combined with elevated LAP, these observed changes following ablation collectively suggest that post-ablation diastolic stunning occurs in the LV. Based on these findings, this study suggests that during the acute phase post-ablation, fluid management is crucial. Fluid intake should be restricted, and patient weight monitoring along with assessment of signs such as lower extremity edema is recommended to prevent acute heart failure. This approach facilitates the timely identification of high-risk patients by clinicians.

## Limitations

First, our study focused exclusively on very early post-ablation echocardiographic changes. The long-term trajectory of systolic functional recovery across all cardiac chambers following CA remains an important area for future investigation. A longitudinal study design with serial follow-up assessments would be particularly valuable for understanding the complete temporal pattern of electromechanical remodeling after ablation. Second, this was a single-center study with a modest sample size. The relatively small sample size and single-center design may limit the generalizability and statistical power of our findings. Future multicenter randomized controlled trials with larger sample sizes are warranted to confirm and extend our observations. Third, the irrigated fluid-induced volume overload may have served as a confounding factor for elevated atrial pressure in this study. Monitoring volume parameters, such as central venous pressure during the procedure, would allow for a more accurate assessment of the impact of volume status on atrial pressure changes.

## Conclusion

This prospective study provides a comprehensive hemodynamic characterization of very early cardiac changes following CA for AF, incorporating biatrial pressure measurements and multimodality imaging assessment. Our findings suggest that systolic functional recovery of all cardiac chambers begins within 48 h post-ablation, with mechanical improvements preceding structural remodeling in AF patients. Furthermore, this study suggests transient left ventricular diastolic dysfunction by multiple hemodynamic parameters. Further mechanistic studies are warranted to elucidate the pathophysiologic processes underlying these dynamic alterations and their clinical implications for post-ablation management.

## Supplementary Information

Supplementary Information 1.

Supplementary Information 2.

Supplementary Information 3.

Supplementary Information 4.

## Data Availability

The data in current study are available from the corresponding author upon reasonable request.

## Abbreviations

AF: Atrial fibrillation
CA: Catheter ablation
LA: Left atrium/atrial
LV: Left ventricle/ventricular
EF: Ejection fraction
RA: Right atrium/atrial
RV: Right ventricle/ventricular
LAP: Left atrial pressure
RAP: Right atrial pressure
IVC: Inferior vena cava
GLS: Global longitudinal strain
Sr: Reservoir strain
Scd: Conduit strain
Sct: Contractile strain
MV: Mitral valve
TV: Tricuspid valve

## References

[1] Joglar, J. A. et al. 2023 ACC/AHA/ACCP/HRS guideline for the diagnosis and management of atrial fibrillation: A report of the American College of Cardiology/American Heart Association Joint Committee on Clinical Practice Guidelines. *Circulation* **149**, e1–e156 (2024). 38033089 10.1161/CIR.0000000000001193 PMC11095842

[2] Thomas, L. & Abhayaratna, W. P. Left atrial reverse remodeling: mechanisms, evaluation, and clinical significance. *JACC Cardiovasc. Imaging* **10**, 65–77 (2017). 28057220 10.1016/j.jcmg.2016.11.003

[3] Sheng, Y. et al. Deciphering mechanisms of cardiomyocytes and non-cardiomyocyte transformation in myocardial remodeling of permanent atrial fibrillation. *J. Adv. Res.* **61**, 101–117 (2024). 37722560 10.1016/j.jare.2023.09.012 PMC11258668

[4] Kim, I. S. et al. The ratio of early transmitral flow velocity (E) to early mitral annular velocity (Em) predicts improvement in left ventricular systolic and diastolic function 1 year after catheter ablation for atrial fibrillation. *Europace* **17**, 1051–1058 (2015). 25600764 10.1093/europace/euu346

[5] Minamisaka, T. et al. Transient manifestation of left ventricular diastolic dysfunction following ablation in patients with paroxysmal atrial fibrillation. *J. Arrhythm.* **41**, 978–984 (2018). 10.1002/clc.22990 PMC6489816 29869416

[6] Koike, T. et al. Prognostic significance of diastolic dysfunction in patients with systolic dysfunction undergoing atrial fibrillation ablation. *Int. J. Cardiol. Heart Vasc.* **41**, 101079 (2022). 35812132 10.1016/j.ijcha.2022.101079 PMC9260613

[7] Andrade, J. G. et al. Progression of atrial fibrillation after cryoablation or drug therapy. *N. Engl. J. Med.* **388**, 105–116 (2023). 36342178 10.1056/NEJMoa2212540

[8] Joglar, J. A. et al. 2023 ACC/AHA/ACCP/HRS Guideline for the Diagnosis and Management of Atrial Fibrillation: A report of the American College of Cardiology/American Heart Association Joint Committee on Clinical Practice Guidelines. *J. Am. Coll. Cardiol.* **83**, 109–279 (2024). 38043043 10.1016/j.jacc.2023.08.017 PMC11104284

[9] Sugumar, H. et al. A prospective STudy using invAsive haemodynamic measurements foLLowing catheter ablation for AF and early HFpEF: STALL AF-HFpEF. *Eur. J. Heart Fail.* **23**, 785–796 (2021). 33565197 10.1002/ejhf.2122

[10] Chieng, D. et al. Atrial fibrillation ablation for heart failure with preserved ejection fraction: A randomized controlled trial. *JACC Heart Fail.* **11**, 646–658 (2023). 36868916 10.1016/j.jchf.2023.01.008

[11] January, C. T. et al. 2019 AHA/ACC/HRS focused update of the 2014 AHA/ACC/HRS guideline for the management of patients with atrial fibrillation: A report of the American College of Cardiology/American Heart Association Task Force on Clinical Practice Guidelines and the Heart Rhythm Society. *J. Am. Coll. Cardiol.* **74**, 104–132 (2019). 30703431 10.1016/j.jacc.2019.01.011

[12] Zhang, X., Wei, M., Xue, P. & Tang, B. Meta-analysis of comprehensive prognostic evaluation in patients with atrial fibrillation complicated by heart failure after catheter ablation. *Sci. Rep.* **15**, 32785 (2025). 40998847 10.1038/s41598-025-16166-3 PMC12464206

[13] Hopman, L. et al. Atrial ablation lesion evaluation by cardiac magnetic resonance: Review of imaging strategies and histological correlations. *JACC Clin. Electrophysiol.* **9**, 2665–2679 (2023). 37737780 10.1016/j.jacep.2023.08.013

[14] Guttman, M. A. et al. Acute enhancement of necrotic radio-frequency ablation lesions in left atrium and pulmonary vein ostia in swine model with non-contrast-enhanced T1-weighted MRI. *Magn. Reson. Med.* **83**, 1368–1379 (2020). 31565818 10.1002/mrm.28001 PMC6949368

[15] Khan, H. R., Yakupoglu, H. Y. & Kralj-Hans, I. Left atrial function predicts atrial arrhythmia recurrence following ablation of long-standing persistent atrial fibrillation. *J. Am. Heart Assoc.* **12**, e015352 (2023). 10.1161/CIRCIMAGING.123.015352 PMC10281195 37288553

[16] Soulat-Dufour, L. et al. Restoring sinus rhythm reverses cardiac remodeling and reduces valvular regurgitation in patients with atrial fibrillation. *J. Am. Coll. Cardiol.* **79**, 951–961 (2022). 35272799 10.1016/j.jacc.2021.12.029

[17] Olsen, F. J. et al. Left atrial structure and function among different subtypes of atrial fibrillation: An echocardiographic substudy of the AMIO-CAT trial. *Eur. Heart J. Cardiovasc. Imaging* **21**, 1386–1394 (2020). 32783051 10.1093/ehjci/jeaa222

[18] Liżewska-Springer, A. et al. Echocardiographic assessment in patients with atrial fibrillation (AF) and normal systolic left ventricular function before and after catheter ablation: If AF begets AF, does pulmonary vein isolation terminate the vicious circle?. *Cardiol. J.* **27**, 126–135 (2020). 30701515 10.5603/CJ.a2019.0004 PMC8016037

[19] Lam, C. S. et al. Atrial fibrillation in heart failure with preserved ejection fraction: Association with exercise capacity, left ventricular filling pressures, natriuretic peptides, and left atrial volume. *JACC Heart Fail.* **5**, 92–98 (2017). 28017355 10.1016/j.jchf.2016.10.005

[20] Calkins, H. et al. 2017 HRS/EHRA/ECAS/APHRS/SOLAECE expert consensus statement on catheter and surgical ablation of atrial fibrillation. *Heart Rhythm* **14**, e275–e444 (2017). 28506916 10.1016/j.hrthm.2017.05.012 PMC6019327

[21] Nagueh, S. F. et al. Recommendations for the evaluation of left ventricular diastolic function by echocardiography: An update from the American Society of Echocardiography and the European Association of Cardiovascular Imaging. *J. Am. Soc. Echocardiogr.* **29**, 277–314 (2016). 27037982 10.1016/j.echo.2016.01.011

[22] Lang, R. M. et al. Recommendations for cardiac chamber quantification by echocardiography in adults: An update from the American Society of Echocardiography and the European Association of Cardiovascular Imaging. *Eur. Heart J. Cardiovasc. Imaging* **16**, 233–270 (2015). 25712077 10.1093/ehjci/jev014

[23] Mukherjee, M. et al. Guidelines for the echocardiographic assessment of the right heart in adults and special considerations in pulmonary hypertension: Recommendations from the American Society of Echocardiography. *J. Am. Soc. Echocardiogr.* **38**, 141–186 (2025). 40044341 10.1016/j.echo.2025.01.006

[24] Badano, L. P. et al. Standardization of left atrial, right ventricular, and right atrial deformation imaging using two-dimensional speckle tracking echocardiography: A consensus document of the EACVI/ASE/Industry Task Force to standardize deformation imaging. *Eur. Heart J. Cardiovasc. Imaging* **19**, 591–600 (2018). 29596561 10.1093/ehjci/jey042

[25] Seo, J. & Yu, H. T. Novel algorithm for non-invasive estimation of left atrial pressure in patients with atrial fibrillation. *Europace* **26**, 414–421 (2025). 10.1093/ehjci/jeae311 39704179

[26] Park, J. S. & Cho, I. Differentiating left atrial pressure responses in paroxysmal and persistent atrial fibrillation: implications for diagnosing heart failure with preserved ejection fraction and managing atrial fibrillation. *J. Am. Heart Assoc.* **13**, e035246 (2024). 39189473 10.1161/JAHA.124.035246 PMC11646497

[27] Lim, H. S. et al. Time course of inflammation, myocardial injury, and prothrombotic response after radiofrequency catheter ablation for atrial fibrillation. *Circ. Arrhythm. Electrophysiol.* **7**, 83–89 (2014). 24446024 10.1161/CIRCEP.113.000876

[28] Ollitrault, P. & Chaumont, C. Superior vena cava isolation using a pentaspline pulsed-field ablation catheter: feasibility and safety in patients undergoing atrial fibrillation catheter ablation. *Europace* **26**, euae149 (2024). 38875490 10.1093/europace/euae160 PMC11252500

[29] Sparks, P. B. et al. Left atrial “stunning” following radiofrequency catheter ablation of chronic atrial flutter. *J. Am. Coll. Cardiol.* **32**, 468–475 (1998). 9708477 10.1016/s0735-1097(98)00253-8

[30] Ariyaratnam, J. P. et al. Identification of subclinical heart failure with preserved ejection fraction in patients with symptomatic atrial fibrillation. *JACC Heart Fail.* **11**, 1626–1638 (2023). 37676212 10.1016/j.jchf.2023.07.019

[31] Hirose, K. & Nakanishi, K. Association of atrial fibrillation progression with left atrial functional reserve and its reversibility. *J. Am. Heart Assoc.* **13**, e032215 (2024). 38156556 10.1161/JAHA.123.032215 PMC10863802

[32] Collier, P., Phelan, D. & Klein, A. A test in context: myocardial strain measured by speckle-tracking echocardiography. *J. Am. Coll. Cardiol.* **69**, 1043–1056 (2017). 28231932 10.1016/j.jacc.2016.12.012

[33] Li, Y. et al. Left atrial strain for predicting recurrence in patients with non-valvular atrial fibrillation after catheter ablation: A single-center two-dimensional speckle tracking retrospective study. *BMC Cardiovasc. Disord.* **22**, 468 (2022). 36335294 10.1186/s12872-022-02916-y PMC9637312

[34] Kerut, E. K., McIlwain, E. & Nishimura, R. A. Grade I diastolic dysfunction and elevated left ventricular end-diastolic pressure: Mitral Doppler inflow, pulmonary vein atrial reversal, and the M-mode mitral B-bump. *Echocardiography* **34**, 1371–1373 (2017). 28737016 10.1111/echo.13631

[35] You, L. et al. Effects of different ablation strategies on long-term left atrial function in patients with paroxysmal atrial fibrillation: A single-blind randomized controlled trial. *Sci. Rep.* **9**, 7695 (2019). 31118449 10.1038/s41598-019-44168-5 PMC6531434

[36] Shechter, A. & Butcher, S. C. The prognostic value of pulmonary venous flow reversal in patients with significant degenerative mitral regurgitation. *Struct. Heart* **10**, 100253 (2023). 10.3390/jcdd10020049 PMC9965059 36826545

[37] Mukai, Y. & Nakanishi, K. Prevalence, associated factors, and echocardiographic estimation of left atrial hypertension in patients with atrial fibrillation. *J. Am. Heart Assoc.* **12**, e030325 (2023). 37702280 10.1161/JAHA.123.030325 PMC10547270

[38] Nakanishi, K. & Daimon, M. Prevalence of glucose metabolism disorders and its association with left atrial remodelling before and after catheter ablation in patients with atrial fibrillation. *Europace* **25**, euad152 (2023). 37155360 10.1093/europace/euad119 PMC10228626

[39] Hermans, A. N. L., Andrade, J. G. & Linz, D. Arterial stiffness association with symptom burden in patients with atrial fibrillation: Direct cause or marker of concomitant risk factors?. *Can. J. Cardiol.* **36**, 1843–1846 (2020). 32810580 10.1016/j.cjca.2020.08.003

[40] Zhang, J. Q. et al. Left ventricular synchronization and systolic function estimated by speckle tracking echocardiography pre- and post-radiofrequency ablation in patients with atrial fibrillation. *Int. J. Cardiol.* **172**, 217–219 (2014). 24485225 10.1016/j.ijcard.2013.12.081

[41] Saijo, Y. et al. Impact of left atrial strain mechanics on exercise intolerance and need for septal reduction therapy in hypertrophic cardiomyopathy. *Eur. Heart J. Cardiovasc. Imaging* **23**, 238–245 (2022). 33462591 10.1093/ehjci/jeab001

[42] Skaarup, K. G. et al. Age- and sex-based normal values of layer-specific longitudinal and circumferential strain by speckle tracking echocardiography: The Copenhagen City Heart Study. *Eur. Heart J. Cardiovasc. Imaging* **23**, 629–640 (2022). 33624014 10.1093/ehjci/jeab032

[43] Kim, M. & Uhm, J. S. The effects of radiofrequency catheter ablation for atrial fibrillation on right ventricular function. *J. Cardiovasc. Electrophysiol.* **35**, 203–217 (2024). 10.4070/kcj.2023.0312 PMC11040267 38654567

