# Supplementary material for: Very early cardiac hemodynamic changes after atrial fibrillation ablation
Source: Sci Rep. 2025 Dec 29;15:44759. doi: 10.1038/s41598-025-28591-5 (PMC12749351; doi:10.1038/s41598-025-28591-5)
Supplement: Supplementary file 1 — Supplementary Information 1. [file 41598_2025_28591_MOESM1_ESM.docx]

**Figure S1. Left atrial pressure (LAP) and right atrial pressure (RAP) measurements during ablation.**

The red line indicates LAP (right panel) and RAP (left panel), including maximum, minimum, and mean values (numerical values adjacent to the red line).

**Figure S2. Early recovery of left atrial and left ventricular systolic function post-ablation.**

Left atrial (LA) functional parameters (upper and lower left panels) showed post-ablation improvements in LA reservoir strain, LA conduit strain, LA ejection fraction. Left ventricular systolic function, assessed by global longitudinal strain (GLS), also improved (upper and lower right panels).

**Figure S3. Early recovery of right atrial and right ventricular systolic function post-ablation.**

Right atrial (RA) functional parameters (upper and lower left panels) showed post-ablation improvements in RA reservoir strain, RA conduit strain, RA ejection fraction. Left ventricular systolic function, assessed by tricuspid annular plane systolic excursion (TAPSE), also improved (upper and lower right panels).
