# Supplementary figures and images for: Very early cardiac hemodynamic changes after atrial fibrillation ablation
Source: Sci Rep. 2025 Dec 29;15:44759. doi: 10.1038/s41598-025-28591-5 (PMC12749351; doi:10.1038/s41598-025-28591-5)

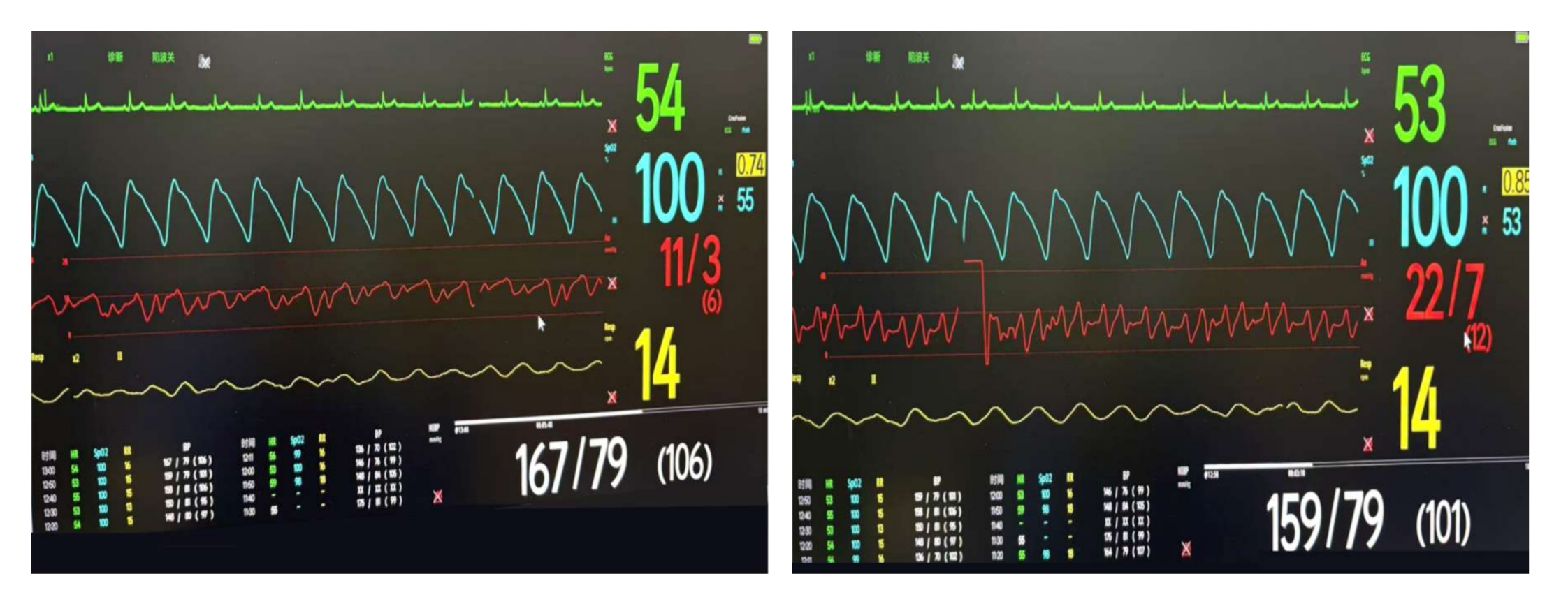

Supplement: Supplementary file 2 — Supplementary Information 2. [file 41598_2025_28591_MOESM2_ESM.tif]

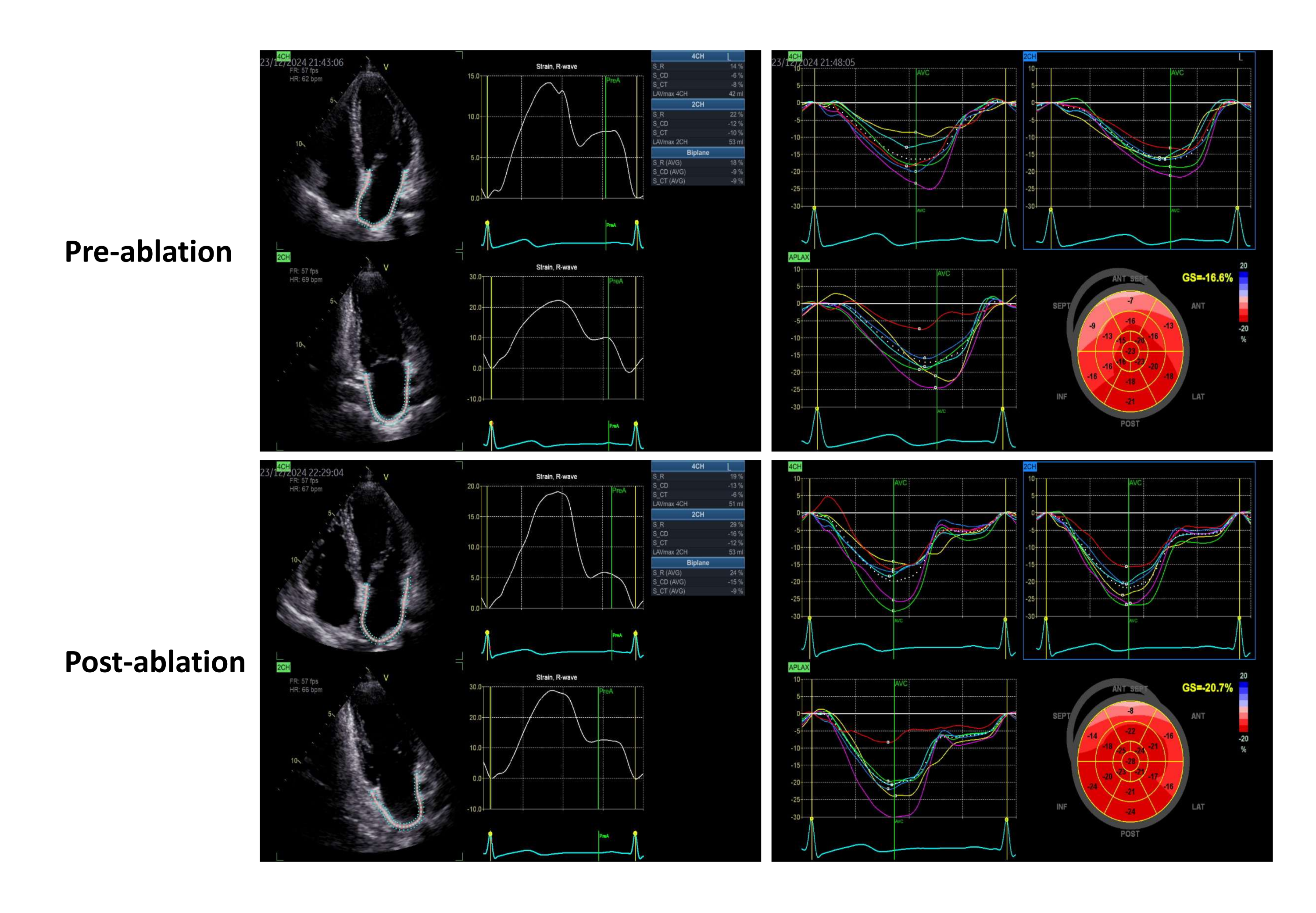

Supplement: Supplementary file 3 — Supplementary Information 3. [file 41598_2025_28591_MOESM3_ESM.tif]

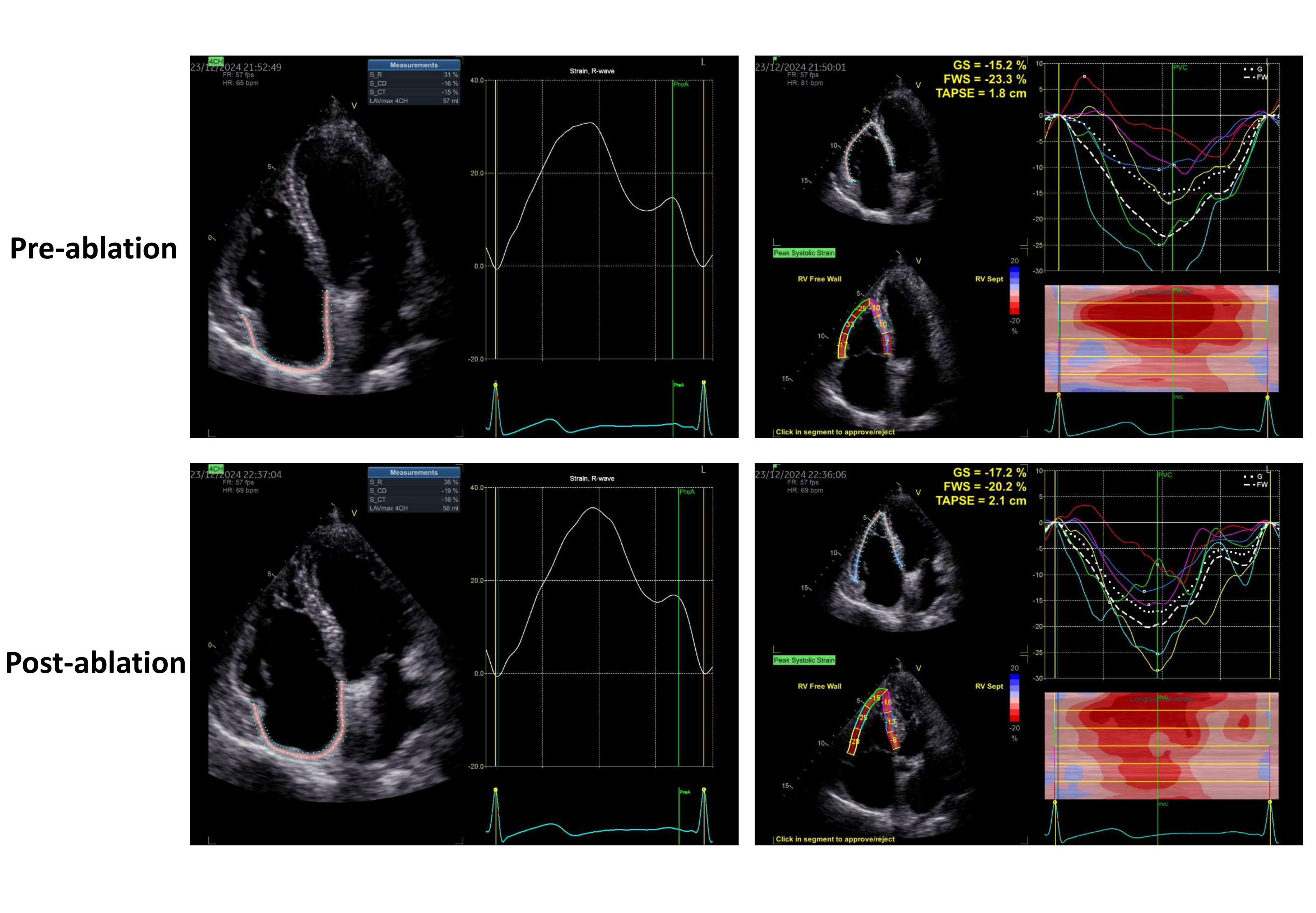

Supplement: Supplementary file 4 — Supplementary Information 4. [file 41598_2025_28591_MOESM4_ESM.tif]
